# Supplementary material for: fhl2b mediates extraocular muscle protection in zebrafish models of muscular dystrophies and its ectopic expression ameliorates affected body muscles
Source: Nat Commun. 2024 Mar 2;15:1950. doi: 10.1038/s41467-024-46187-x (PMC10908798; doi:10.1038/s41467-024-46187-x)
Supplement: Supplementary file 1 — Supplementary Information [file 41467_2024_46187_MOESM1_ESM.pdf]

Supplementary Information

fhl2b expression ameliorates muscular dystrophy

Nils Dennhag[1, 2], Abraha Kahsay[1, 2], Itzel Nissen[3, 4], Hanna Nord[1], Maria Chermenina[1, 2], Jiao Liu[5,6], Anders Arner[5], Jing-Xia Liu[1], Ludvig J Backman[1], Silvia Remeseiro[3,4], Jonas von Hofsten[1*] and Fatima Pedrosa Domellöf[1, 4*].

[1]Department of Medical and Translational Biology, Umeå University, Sweden

[2]Department of Clinical Sciences, Ophthalmology, Umeå University, Sweden

[3]Department of Medical and Translational Biology; Section of Molecular Medicine, Umeå University, Sweden

[4]Wallenberg Center for Molecular Medicine (WCMM), Umeå University, Sweden

[5]Div. Thoracic Surgery, Dept. Clinical Sciences, Lund University, Lund, Sweden

[6]College of Life Sciences, South-Central University for Nationalities, Wuhan, China

*These authors jointly supervised this work.

Correspondence should be addressed to Jonas von Hofsten (Jonas.von.hofsten@umu.se) or Fatima Pedrosa Domellöf (fatima.pedrosa-domellof@umu.se).

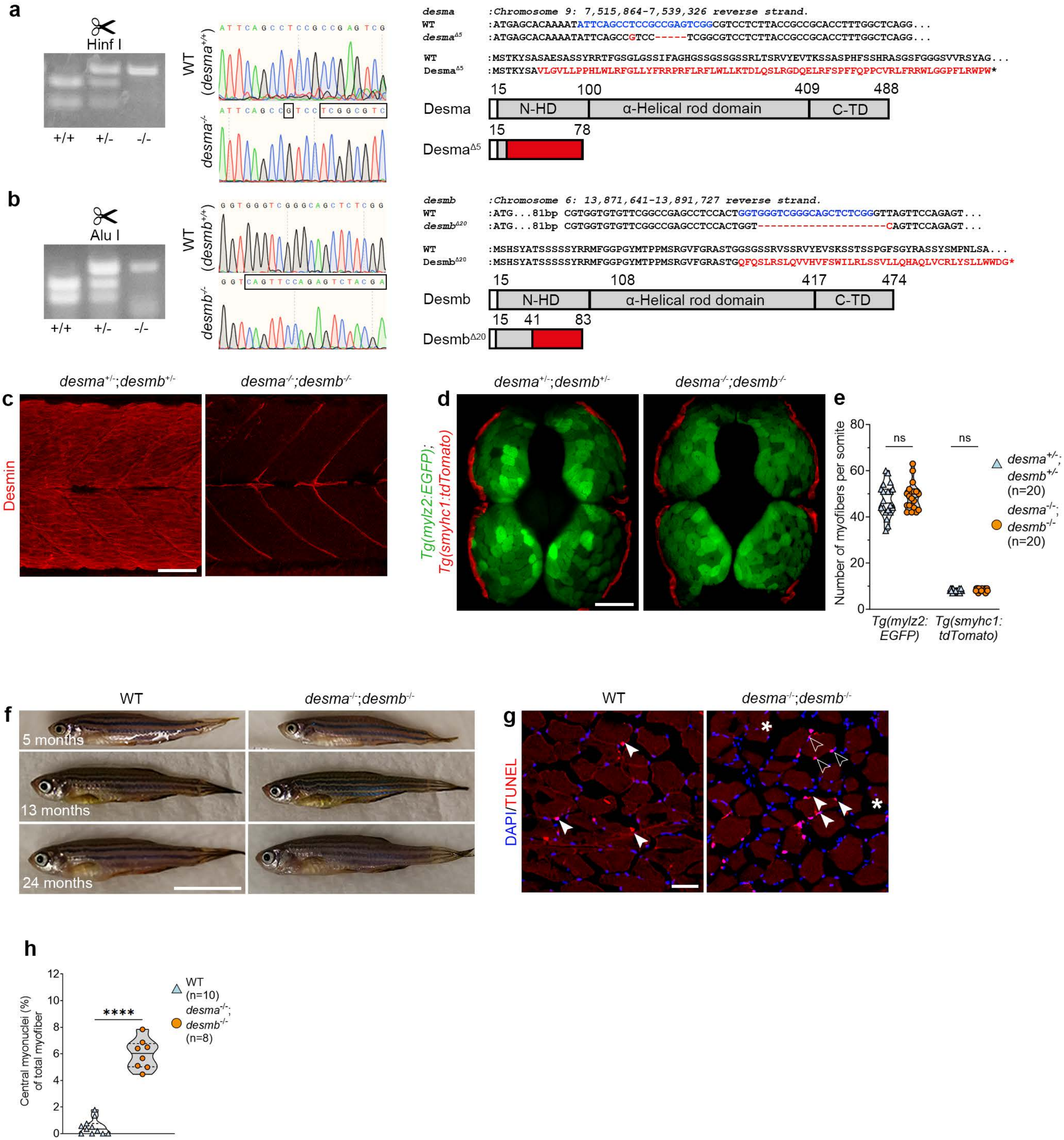
a
Hinf I
+/+ +/- -/-
WT (desma+/+)
desma-/-
desma :Chromosome 9: 7,515,864-7,539,326 reverse strand.
WT :ATGAGCACAAAATATTCAGCCTCCGCCGAGTCGGCGTCCTCTTACCGCCGCACCTTTGGCTCAGG...
desmaΔ5 :ATGAGCACAAAATATTCAGCCGTCC-----TCGGCGTCCTCTTACCGCCGCACCTTTGGCTCAGG...
WT :MSTKYSASAESASSYRRTFGSGLGSSIFAGHGSSGSSGSSRLTSRVYEVTKSSASPHFSSHRASGSFGGGSVVRSYAG...
DesmaΔ5 :MSTKYSAVLGVLLPPHLWLRFGLLYFRRPRFLRFLWLLKTDLQSLRGDQELRFSPFFQPPCVRLFRRWLGGPFLRWPW*
15 100 409 488
Desma N-HD α-Helical rod domain C-TD
15 78
DesmaΔ5
b
Alu I
+/+ +/- -/-
WT (desmb+/+)
desmb-/-
desmb :Chromosome 6: 13,871,641-13,891,727 reverse strand.
WT :ATG...81bp CGTGGTGTGTTCGGCCGAGCCTCCACTGGTGGGTCGGGCAGCTCTCGGGTTAGTTCCAGAGT...
desmbΔ20 :ATG...81bp CGTGGTGTGTTCGGCCGAGCCTCCACTGGT--------------------CAGTTCCAGAGT...
WT :MSHSYATSSSSSYRRMFGGPGYMTPPMSRGVFGRASTGGSGSSRVSSRVYEVSKSSTSSPGFSGYRASSYSMPNLSA...
DesmbΔ20 :MSHSYATSSSSSYRRMFGGPGYMTPPMSRGVFGRASTGQFQSLRSLQVVHVFSWILRLSSVLLQHAQLVCRLYSLLWWDG*
15 108 417 474
Desmb N-HD α-Helical rod domain C-TD
15 41 83
DesmbΔ20
c
desma+/-;desmb+/-
desma-/-;desmb-/-
Desmin
d
desma+/-;desmb+/-
desma-/-;desmb-/-
Tg(mylz2:EGFP); Tg(smyhc1:tdTomato)
e
Number of myofibers per somite
ns
ns
Tg(mylz2: EGFP)
Tg(smyhc1: tdTomato)
desma+/-; desmb+/- (n=20)
desma-/-; desmb-/- (n=20)
f
WT
desma-/-;desmb-/-
5 months
13 months
24 months
g
WT
desma-/-;desmb-/-
DAPI/TUNEL
h
Central myonuclei (%) of total myofiber
****
WT (n=10)
desma-/-; desmb-/- (n=8)

Supplemental Figure 1. Lack of desmin does not cause decreased myofiber numbers or impaired growth. a) Generation of *desma* and **b)** *desmb* CRISPR/Cas9 mediated knockout. Blue text shows CRISPR target site and red text shows deletions and faulty amino acids. Asterisk indicates a premature stop codon. **c)** Desmin immunolabeling in *desma*$^{-/-}$*;desmb*$^{-/-}$ mutants and *desma*$^{+/-}$*;desmb*$^{+/-}$ sibling control larvae at 3 dpf. **d)** *Tg(mylz2:EGFP)* positive fast (green) and *Tg(smyhc1:tdTomato)* positive slow (red) myofibers at 5 dpf in *desma*$^{+/-}$*;desmb*$^{+/-}$ control and *desma*$^{-/-}$*;desmb*$^{-/-}$ larvae trunk cross section. **e)** Quantification of *Tg(mylz2:EGFP)* positive fast and *Tg(smyhc1:tdTomato)* positive slow myofibers in *desma*$^{-/-}$*;desmb*$^{-/-}$ mutants and *desma*$^{+/-}$*;desmb*$^{+/-}$ sibling control larvae at 5 dpf. **f)** Adult *desma*$^{-/-}$*; desmb*$^{-/-}$ and WT (*desma*$^{+/+}$*;desmb*$^{+/+}$) controls at 5, 13 and 24 months. **g)** Cross-sections of WT and *desma*$^{-/-}$*;desmb*$^{-/-}$ 24 months old zebrafish trunk fast domain muscle labeled for DAPI/TUNEL. Arrowheads indicates TUNEL/DAPI double positive nuclei. Open arrowheads indicates TUNEL/DAPI positive nuclei on a different myofiber as compared to closed arrowheads. Asterisk indicate myofibers with central nuclei. **h)** Quantification of myofibers with central nuclei in the fast domain of 24 months old zebrafish trunks showed significantly more regenerating myofibers in *desma*$^{-/-}$*; desmb*$^{-/-}$ as compared to WT controls ($p=2.1e^{-7}$). Statistical analysis in e, h: Two-sided t-tests with Welch correction. Data in violin plots is presented as median (line) and quartiles (dashed line). Scale bar in c, d, g: 50 µm, f: 1 cm. Schematic images were adapted from https://www.biorender.com.

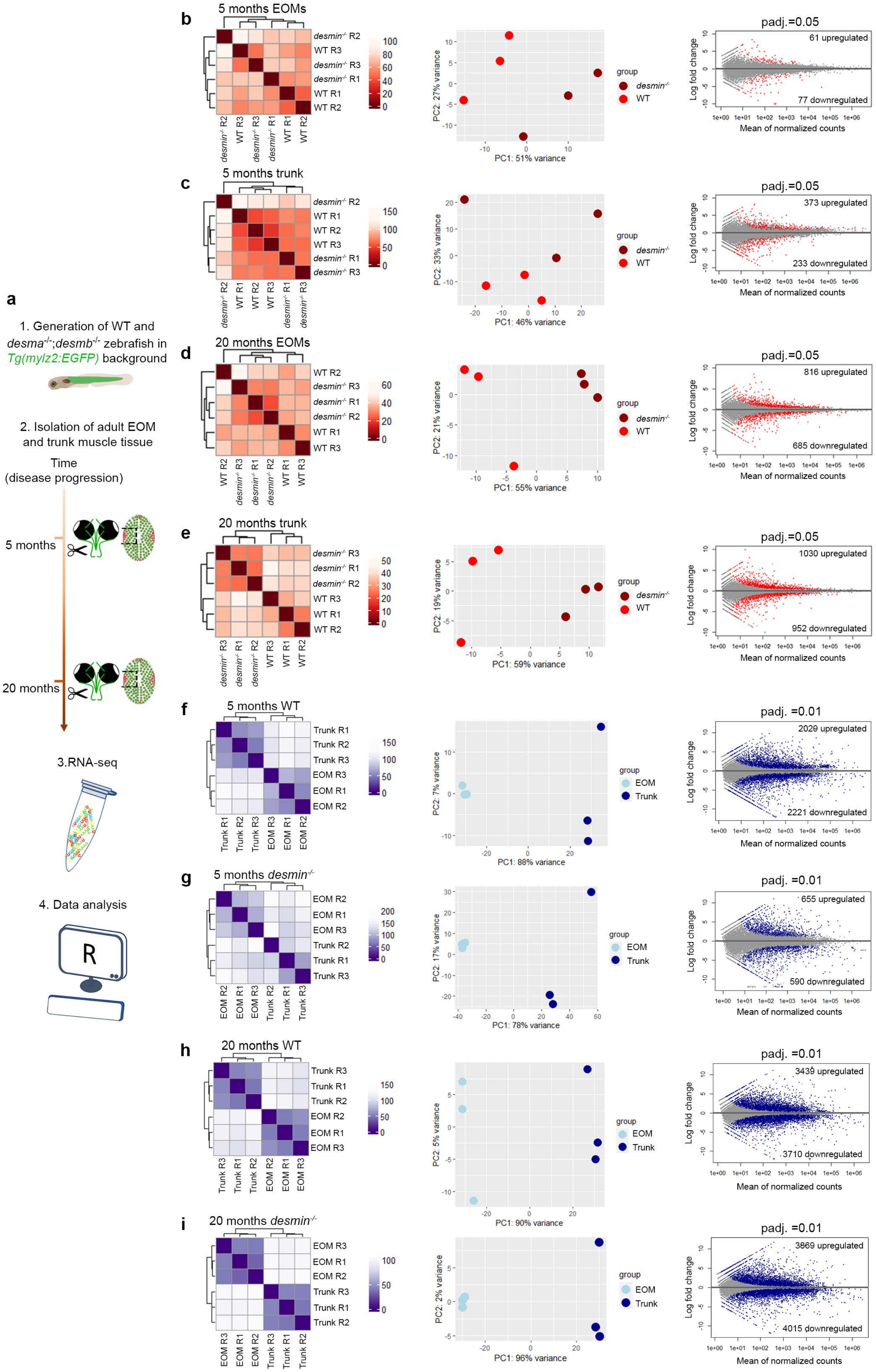

a
1. Generation of WT and
desma-/-;desmb-/- zebrafish in
Tg(mylz2:EGFP) background
2. Isolation of adult EOM
and trunk muscle tissue
Time
(disease progression)
5 months
20 months
3.RNA-seq
4. Data analysis
R
b
5 months EOMs
c
5 months trunk
d
20 months EOMs
e
20 months trunk
f
5 months WT
g
5 months desmin-/-
h
20 months WT
i
20 months desmin-/-
group
desmin-/-
WT
EOM
Trunk
PC1: 51% variance
PC2: 27% variance
PC1: 46% variance
PC2: 33% variance
PC1: 55% variance
PC2: 21% variance
PC1: 59% variance
PC2: 19% variance
PC1: 88% variance
PC2: 7% variance
PC1: 78% variance
PC2: 17% variance
PC1: 90% variance
PC2: 5% variance
PC1: 96% variance
PC2: 2% variance
padj.=0.05
61 upregulated
77 downregulated
373 upregulated
233 downregulated
816 upregulated
685 downregulated
1030 upregulated
952 downregulated
padj. =0.01
2029 upregulated
2221 downregulated
655 upregulated
590 downregulated
3439 upregulated
3710 downregulated
3869 upregulated
4015 downregulated
Log fold change
Mean of normalized counts

Supplemental Figure 2. Transcriptomic profiling of extraocular and trunk muscles in five and 20 months old zebrafish. a) Workflow for RNA-sequencing of 5 months and 20 months old WT (*desma*$^{+/+}$*; desmb*$^{+/+}$) and *desma*$^{-/-}$*; desmb*$^{-/-}$ zebrafish larvae in *Tg(mylz2:EGFP)* background to visualize muscle for collection of tissue. **b-i)** Differential expression analysis performed for different pairwise comparisons: **b)** 5 months old EOMs in *desma*$^{-/-}$*;desmb*$^{-/-}$ *vs* WT, **c)** 5 months old trunk muscle in *desma*$^{-/-}$*;desmb*$^{-/-}$ *vs* WT, **d)** 20 months old EOMs in *desma*$^{-/-}$*;desmb*$^{-/-}$ *vs* WT, **e)** 20 months old trunk muscle in *desma*$^{-/-}$*;desmb*$^{-/-}$ *vs* WT, **f)** 5 months old EOMs *vs* trunk muscle in WT, **g)** 5 months old EOMs *vs* trunk muscle in *desma*$^{-/-}$*;desmb*$^{-/-}$, **h)** 20 months old EOMs *vs* trunk muscle in WT and **i)** 20 months old EOMs *vs* trunk muscle in *desma*$^{-/-}$*;desmb*$^{-/-}$ zebrafish. Sample-to-simple distance heatmaps (left in b-i), PCA plots (middle in b-i) and MA plots (right in b-i) are displayed. MA plots show genes differentially expressed (DEGs, colored dots) for each comparison. Schematic images were adapted from https://www.biorender.com.

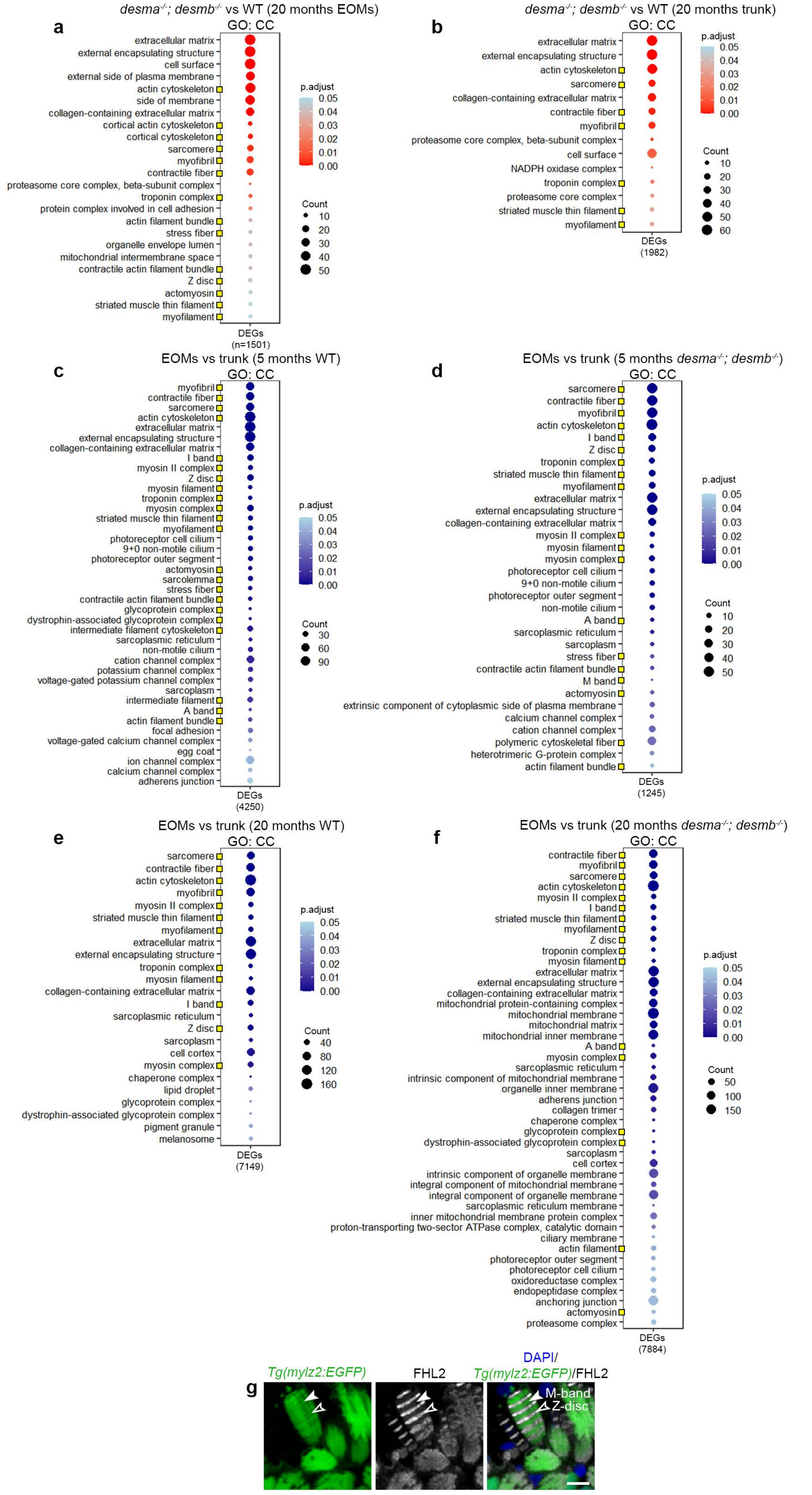
a
desma-/-; desmb-/- vs WT (20 months EOMs)
GO: CC
extracellular matrix
external encapsulating structure
cell surface
external side of plasma membrane
actin cytoskeleton
side of membrane
collagen-containing extracellular matrix
cortical actin cytoskeleton
cortical cytoskeleton
sarcomere
myofibril
contractile fiber
proteasome core complex, beta-subunit complex
troponin complex
protein complex involved in cell adhesion
actin filament bundle
stress fiber
organelle envelope lumen
mitochondrial intermembrane space
contractile actin filament bundle
Z disc
actomyosin
striated muscle thin filament
myofilament
DEGs
(n=1501)
p.adjust
0.05
0.04
0.03
0.02
0.01
0.00
Count
10
20
30
40
50
b
desma-/-; desmb-/- vs WT (20 months trunk)
GO: CC
extracellular matrix
external encapsulating structure
actin cytoskeleton
sarcomere
collagen-containing extracellular matrix
contractile fiber
myofibril
proteasome core complex, beta-subunit complex
cell surface
NADPH oxidase complex
troponin complex
proteasome core complex
striated muscle thin filament
myofilament
DEGs
(1982)
p.adjust
0.05
0.04
0.03
0.02
0.01
0.00
Count
10
20
30
40
50
60
c
EOMs vs trunk (5 months WT)
GO: CC
myofibril
contractile fiber
sarcomere
actin cytoskeleton
extracellular matrix
external encapsulating structure
collagen-containing extracellular matrix
I band
myosin II complex
Z disc
myosin filament
troponin complex
myosin complex
striated muscle thin filament
myofilament
photoreceptor cell cilium
9+0 non-motile cilium
photoreceptor outer segment
actomyosin
sarcolemma
stress fiber
contractile actin filament bundle
glycoprotein complex
dystrophin-associated glycoprotein complex
intermediate filament cytoskeleton
sarcoplasmic reticulum
non-motile cilium
cation channel complex
potassium channel complex
voltage-gated potassium channel complex
sarcoplasm
intermediate filament
A band
actin filament bundle
focal adhesion
voltage-gated calcium channel complex
egg coat
ion channel complex
calcium channel complex
adherens junction
DEGs
(4250)
p.adjust
0.05
0.04
0.03
0.02
0.01
0.00
Count
30
60
90
d
EOMs vs trunk (5 months desma-/-; desmb-/-)
GO: CC
sarcomere
contractile fiber
myofibril
actin cytoskeleton
I band
Z disc
troponin complex
striated muscle thin filament
myofilament
extracellular matrix
external encapsulating structure
collagen-containing extracellular matrix
myosin II complex
myosin filament
myosin complex
photoreceptor cell cilium
9+0 non-motile cilium
photoreceptor outer segment
non-motile cilium
A band
sarcoplasmic reticulum
sarcoplasm
stress fiber
contractile actin filament bundle
M band
actomyosin
extrinsic component of cytoplasmic side of plasma membrane
calcium channel complex
cation channel complex
polymeric cytoskeletal fiber
heterotrimeric G-protein complex
actin filament bundle
DEGs
(1245)
p.adjust
0.05
0.04
0.03
0.02
0.01
0.00
Count
10
20
30
40
50
e
EOMs vs trunk (20 months WT)
GO: CC
sarcomere
contractile fiber
actin cytoskeleton
myofibril
myosin II complex
striated muscle thin filament
myofilament
extracellular matrix
external encapsulating structure
troponin complex
myosin filament
collagen-containing extracellular matrix
I band
sarcoplasmic reticulum
Z disc
sarcoplasm
cell cortex
myosin complex
chaperone complex
lipid droplet
glycoprotein complex
dystrophin-associated glycoprotein complex
pigment granule
melanosome
DEGs
(7149)
p.adjust
0.05
0.04
0.03
0.02
0.01
0.00
Count
40
80
120
160
f
EOMs vs trunk (20 months desma-/-; desmb-/-)
GO: CC
contractile fiber
myofibril
sarcomere
actin cytoskeleton
myosin II complex
I band
striated muscle thin filament
myofilament
Z disc
troponin complex
myosin filament
extracellular matrix
external encapsulating structure
collagen-containing extracellular matrix
mitochondrial protein-containing complex
mitochondrial membrane
mitochondrial matrix
mitochondrial inner membrane
A band
myosin complex
sarcoplasmic reticulum
intrinsic component of mitochondrial membrane
organelle inner membrane
adherens junction
collagen trimer
chaperone complex
glycoprotein complex
dystrophin-associated glycoprotein complex
sarcoplasm
cell cortex
intrinsic component of organelle membrane
integral component of mitochondrial membrane
integral component of organelle membrane
sarcoplasmic reticulum membrane
inner mitochondrial membrane protein complex
proton-transporting two-sector ATPase complex, catalytic domain
ciliary membrane
actin filament
photoreceptor outer segment
photoreceptor cell cilium
oxidoreductase complex
endopeptidase complex
anchoring junction
actomyosin
proteasome complex
DEGs
(7884)
p.adjust
0.05
0.04
0.03
0.02
0.01
0.00
Count
50
100
150
g
Tg(mylz2:EGFP)
FHL2
DAPI/
Tg(mylz2:EGFP)/FHL2
M-band
Z-disc

Supplemental Figure 3. Gene ontology signatures in extraocular and trunk muscles. Gene ontology terms enriched in the DEGs obtained from the comparisons **a)** 20 months old EOMs in *desma*$^{-/-}$;*desmb*$^{-/-}$ *vs* WT, **b)** 20 months old trunk muscle in *desma*$^{-/-}$;*desmb*$^{-/-}$ *vs* WT, **c)** 5 months old EOMs *vs* trunk muscle in WT, **d)** 5 months old EOMs *vs* trunk muscle in *desma*$^{-/-}$;*desmb*$^{-/-}$, **e)** 20 months old EOMs *vs* trunk muscle in WT and **f)** 20 months old EOMs *vs* trunk muscle in *desma*$^{-/-}$;*desmb*$^{-/-}$ zebrafish. Yellow squares indicate myofiber related GO terms selected for further investigation for each comparison. CC: Cellular Compartment. **g)** Fhl2 was found to be present mainly at the Z-disc in EOM myofibers studied using *Tg(mylz2:EGFP)* combined with Fhl2 antibodies and DAPI. Closed arrowheads indicate M-band positioning and open arrowheads indicate Z-disc positioning. Scale bar: 10 µm.

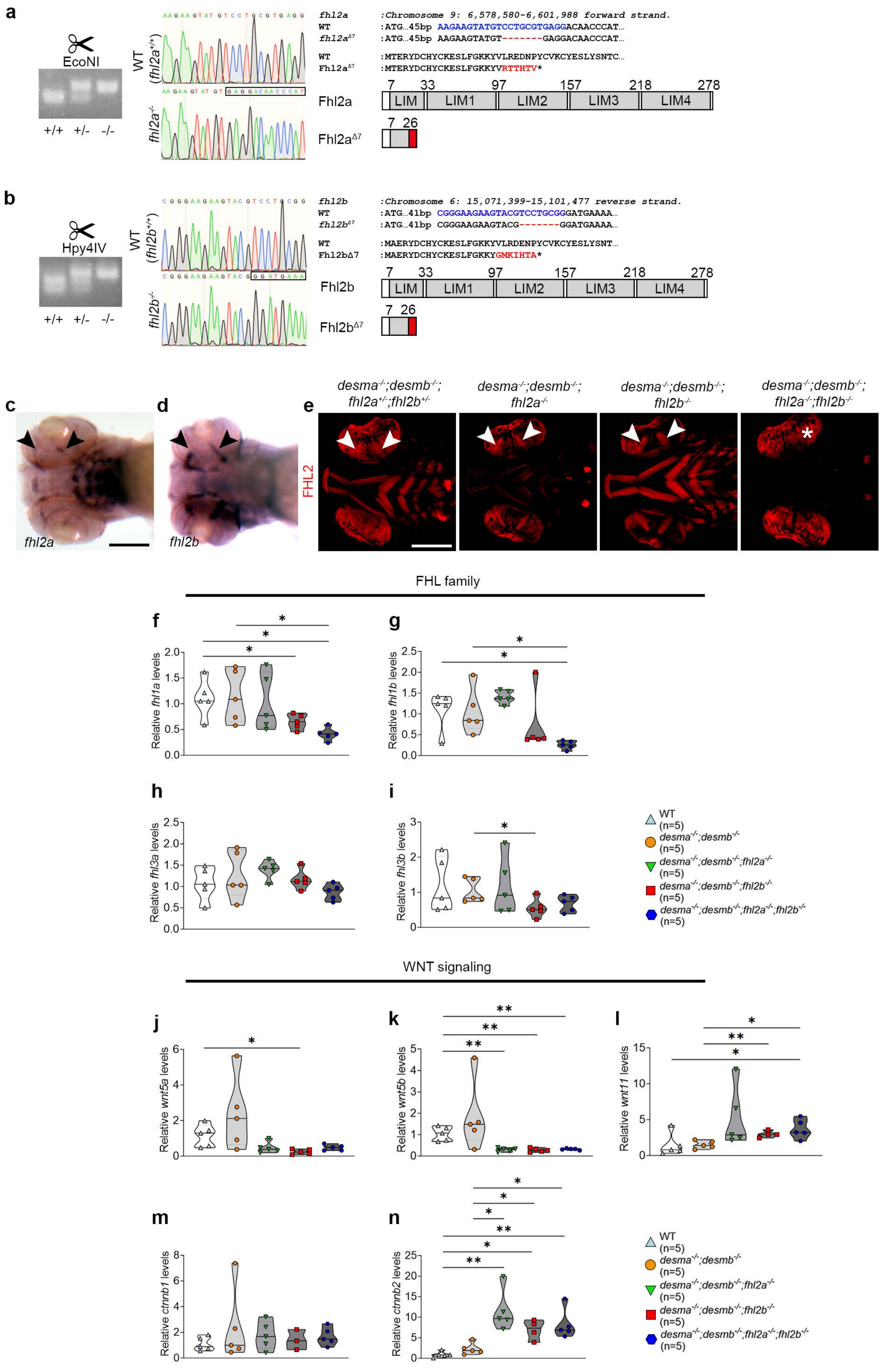

a
EcoNI
+/+ +/- -/-
WT (fhl2a+/+)
fhl2a-/-
fhl2a :Chromosome 9: 6,578,580-6,601,988 forward strand.
WT :ATG…45bp AAGAAGTATGTCCTGCGTGAGGACAACCCAT…
fhl2aΔ7 :ATG…45bp AAGAAGTATGT-------GAGGACAACCCAT…
WT :MTERYDCHYCKESLFGKKYVLREDNPYCVKCYESLYSNTC…
Fhl2aΔ7 :MTERYDCHYCKESLFGKKYVRTTHTV*
Fhl2a
7 33 97 157 218 278
LIM LIM1 LIM2 LIM3 LIM4
Fhl2aΔ7
7 26
b
Hpy4IV
+/+ +/- -/-
WT (fhl2b+/+)
fhl2b-/-
fhl2b :Chromosome 6: 15,071,399-15,101,477 reverse strand.
WT :ATG…41bp CGGGAAGAAGTACGTCCTGCGGGATGAAAA…
fhl2bΔ7 :ATG…41bp CGGGAAGAAGTACG-------GGATGAAAA…
WT :MAERYDCHYCKESLFGKKYVLRDENPYCVKCYESLYSNT…
Fhl2bΔ7 :MAERYDCHYCKESLFGKKYGMKIHTA*
Fhl2b
7 33 97 157 218 278
LIM LIM1 LIM2 LIM3 LIM4
Fhl2bΔ7
7 26
c
fhl2a
d
fhl2b
e
FHL2
desma-/-;desmb-/-; fhl2a+/-;fhl2b+/-
desma-/-;desmb-/-; fhl2a-/-
desma-/-;desmb-/-; fhl2b-/-
desma-/-;desmb-/-; fhl2a-/-;fhl2b-/-
FHL family
f
Relative fhl1a levels
g
Relative fhl1b levels
h
Relative fhl3a levels
i
Relative fhl3b levels
WT (n=5)
desma-/-;desmb-/- (n=5)
desma-/-;desmb-/-;fhl2a-/- (n=5)
desma-/-;desmb-/-;fhl2b-/- (n=5)
desma-/-;desmb-/-;fhl2a-/-;fhl2b-/- (n=5)
WNT signaling
j
Relative wnt5a levels
k
Relative wnt5b levels
l
Relative wnt11 levels
m
Relative ctnnb1 levels
n
Relative ctnnb2 levels
WT (n=5)
desma-/-;desmb-/- (n=5)
desma-/-;desmb-/-;fhl2a-/- (n=5)
desma-/-;desmb-/-;fhl2b-/- (n=5)
desma-/-;desmb-/-;fhl2a-/-;fhl2b-/- (n=5)

Supplemental Figure 4. *fhl2* knockout significantly alters the WNT-signaling pathway. Generation of **a)** *fhl2a* and **b)** *fhl2b* CRISPR/Cas9 mediated knockout zebrafish. Blue text shows CRISPR target site and red text shows deletions and faulty amino acids due to deletions. Asterisk indicates a premature stop codon. *in situ* hybridization of whole mount larvae at 5 dpf showing expression of **c)** *fhl2a* and **d)** *fhl2b.* **e)** Immunolabeling of Fhl2 in *desma*$^{-/-}$;*desmb*$^{-/-}$;*fhl2a*$^{+/-}$;*fhl2b*$^{+/-}$, *desma*$^{-/-}$;*desmb*$^{-/-}$;*fhl2a*$^{-/-}$, *desma*$^{-/-}$;*desmb*$^{-/-}$;*fhl2b*$^{-/-}$ and *desma*$^{-/-}$;*desmb*$^{-/-}$;*fhl2a*$^{-/-}$;*fhl2b*$^{-/-}$ larvae at 5 dpf, asterisk indicates lack of immunolabeling. Arrowheads in c-e) indicate EOMs, ventral view**. f)** Levels of *fhl1a*, **g)** *fhl1b*, **h)** *fhl3a* and **i)** *fhl3b* relative to *β-actin* in WT, *desma*$^{-/-}$;*desmb*$^{-/-}$, *desma*$^{-/-}$;*desmb*$^{-/-}$;*fhl2a*$^{-/-}$, *desma*$^{-/-}$;*desmb*$^{-/-}$;*fhl2b*$^{-/-}$ and *desma*$^{-/-}$;*desmb*$^{-/-}$;*fhl2a*$^{-/-}$;*fhl2b*$^{-/-}$. Significant comparisons in f) were *desma*$^{-/-}$;*desmb*$^{-/-}$;*fhl2b*$^{-/-}$ vs WT (p=0.049), *desma*$^{-/-}$;*desmb*$^{-/-}$;*fhl2a*$^{-/-}$;*fhl2b*$^{-/-}$ vs WT (p=0.011) and *desma*$^{-/-}$;*desmb*$^{-/-}$;*fhl2a*$^{-/-}$;*fhl2b*$^{-/-}$ vs *desma*$^{-/-}$;*desmb*$^{-/-}$ (p=0.0305). Significant comparisons in g) were *desma*$^{-/-}$;*desmb*$^{-/-}$;*fhl2a*$^{-/-}$;*fhl2b*$^{-/-}$ vs WT (p=0.013) and *desma*$^{-/-}$;*desmb*$^{-/-}$;*fhl2a*$^{-/-}$;*fhl2b*$^{-/-}$ vs *desma*$^{-/-}$;*desmb*$^{-/-}$ (p=0.0295). Significant comparisons in i) were *desma*$^{-/-}$;*desmb*$^{-/-}$;*fhl2b*$^{-/-}$ vs *desma*$^{-/-}$;*desmb*$^{-/-}$ (p=0.0387). **j)** Levels of *wnt5a*, **k)** *wnt5b*, **l)** *wnt11* and **m)** *ctnnb1*, **n)** *ctnnb2* relative to *β-actin*. Significant comparisons in k) were *desma*$^{-/-}$;*desmb*$^{-/-}$;*fhl2b*$^{-/-}$ vs WT (p=0.03). Significant comparisons in k) were *desma*$^{-/-}$;*desmb*$^{-/-}$;*fhl2a*$^{-/-}$ vs WT (p=0.0058), *desma*$^{-/-}$;*desmb*$^{-/-}$;*fhl2b*$^{-/-}$ vs WT (p=0.0052) and *desma*$^{-/-}$;*desmb*$^{-/-}$;*fhl2a*$^{-/-}$;*fhl2b*$^{-/-}$ vs WT (p=0.0079). Significant comparisons in l) were *desma*$^{-/-}$;*desmb*$^{-/-}$;*fhl2a*$^{-/-}$;*fhl2b*$^{-/-}$ vs WT (p=0.044), *desma*$^{-/-}$;*desmb*$^{-/-}$;*fhl2b*$^{-/-}$ vs *desma*$^{-/-}$;*desmb*$^{-/-}$ (p=0.0039) and *desma*$^{-/-}$;*desmb*$^{-/-}$;*fhl2a*$^{-/-}$;*fhl2b*$^{-/-}$ vs *desma*$^{-/-}$;*desmb*$^{-/-}$ (p=0.0189). Significant comparisons in n) were *desma*$^{-/-}$;*desmb*$^{-/-}$;*fhl2a*$^{-/-}$ vs WT (p=0.008), *desma*$^{-/-}$;*desmb*$^{-/-}$;*fhl2b*$^{-/-}$ vs WT (p=0.012), *desma*$^{-/-}$;*desmb*$^{-/-}$;*fhl2a*$^{-/-}$;*fhl2b*$^{-/-}$ vs WT (p=0.009), *desma*$^{-/-}$;*desmb*$^{-/-}$;*fhl2a*$^{-/-}$ vs *desma*$^{-/-}$;*desmb*$^{-/-}$ (p=0.012), *desma*$^{-/-}$;*desmb*$^{-/-}$;*fhl2b*$^{-/-}$ vs *desma*$^{-/-}$;*desmb*$^{-/-}$ (p=0.0213) and *desma*$^{-/-}$;*desmb*$^{-/-}$;*fhl2a*$^{-/-}$;*fhl2b*$^{-/-}$ vs *desma*$^{-/-}$;*desmb*$^{-/-}$ (p=0.0181). Gene expression levels f–n were measured by qPCR. Scale bar: 100 µm. Statistical analysis in f-n: Two-sided t-tests with Welch correction. Data in violin plots is presented as median (line) and quartiles (dashed line). Schematic images were adapted from https://www.biorender.com.

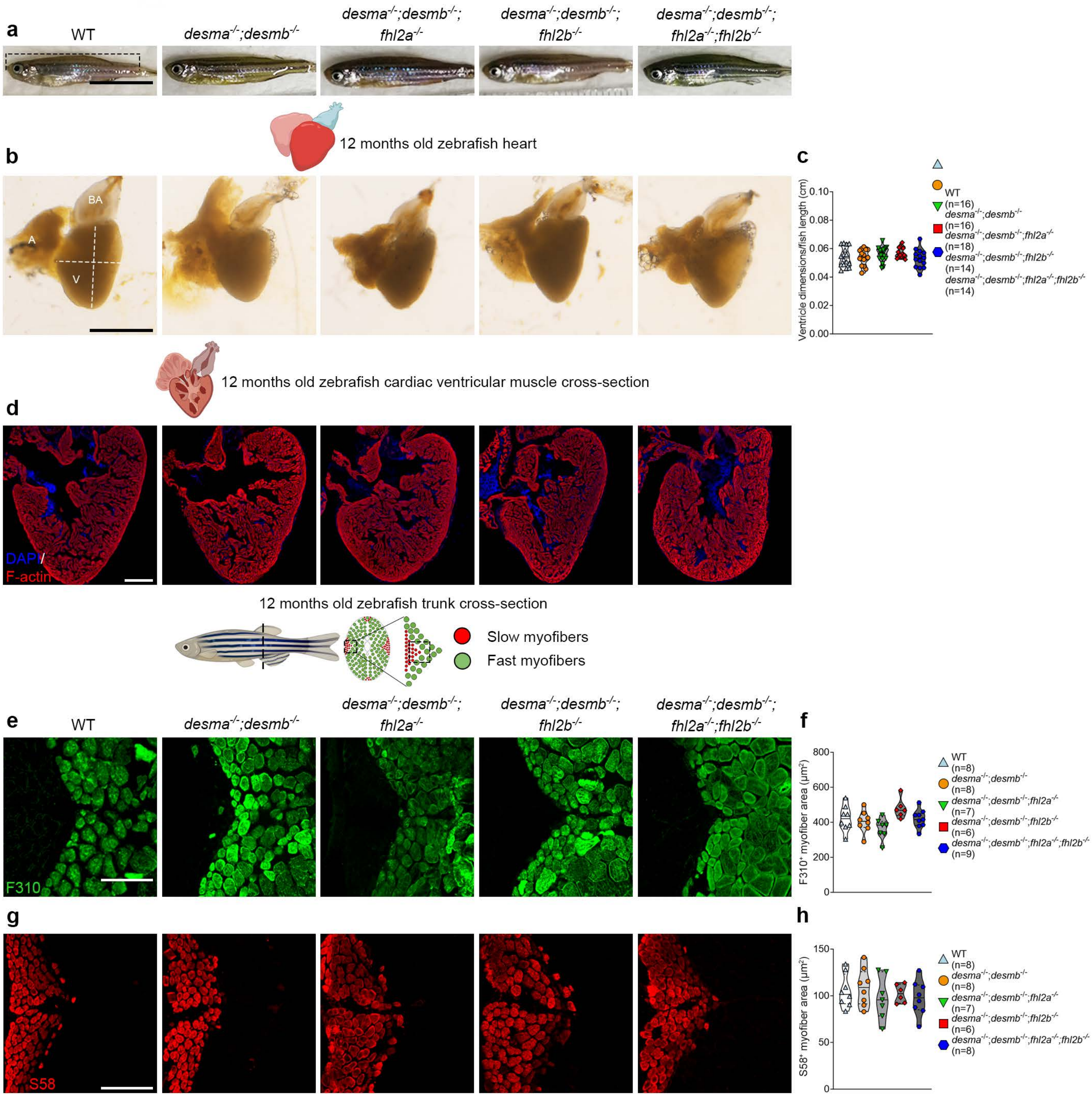

a
WT
desma-/-;desmb-/-
desma-/-;desmb-/-; fhl2a-/-
desma-/-;desmb-/-; fhl2b-/-
desma-/-;desmb-/-; fhl2a-/-;fhl2b-/-
12 months old zebrafish heart
b
BA
A
V
c
Ventricle dimensions/fish length (cm)
WT (n=16)
desma-/-;desmb-/- (n=16)
desma-/-;desmb-/-;fhl2a-/- (n=18)
desma-/-;desmb-/-;fhl2b-/- (n=14)
desma-/-;desmb-/-;fhl2a-/-;fhl2b-/- (n=14)
12 months old zebrafish cardiac ventricular muscle cross-section
d
DAPI/
F-actin
12 months old zebrafish trunk cross-section
Slow myofibers
Fast myofibers
e
f
F310
F310+ myofiber area (μm2)
WT (n=8)
desma-/-;desmb-/- (n=8)
desma-/-;desmb-/-;fhl2a-/- (n=7)
desma-/-;desmb-/-;fhl2b-/- (n=6)
desma-/-;desmb-/-;fhl2a-/-;fhl2b-/- (n=9)
g
h
S58
S58+ myofiber area (μm2)
WT (n=8)
desma-/-;desmb-/- (n=8)
desma-/-;desmb-/-;fhl2a-/- (n=7)
desma-/-;desmb-/-;fhl2b-/- (n=6)
desma-/-;desmb-/-;fhl2a-/-;fhl2b-/- (n=8)

Supplemental Figure 5. Knockout of *desmin* and *fhl2* does not cause cardiac or trunk muscle hypertrophy. a) Lateral view and **b)** dissected heart of WT, *desma*$^{-/-}$*;desmb*$^{-/-}$, *desma*$^{-/-}$*;desmb*$^{-/-}$*;fhl2a*$^{-/-}$, *desma*$^{-/-}$*;desmb*$^{-/-}$*;fhl2b*$^{-/-}$ and *desma*$^{-/-}$*;desmb*$^{-/-}$*;fhl2a*$^{-/-}$*;fhl2b*$^{-/-}$ 12 months old zebrafish. Brackets in a) indicate length of fish, dashed lines in b) indicates ventricle dimensions measured and presented in relation to the length of the fish in **c)**. **d)** Cross section of heart labelled with Phalloidin of WT, *desma*$^{-/-}$*;desmb*$^{-/-}$, *desma*$^{-/-}$*;desmb*$^{-/-}$*;fhl2a*$^{-/-}$, *desma*$^{-/-}$*;desmb*$^{-/-}$*;fhl2b*$^{-/-}$ and *desma*$^{-/-}$*;desmb*$^{-/-}$*;fhl2a*$^{-/-}$*;fhl2b*$^{-/-}$ 12 months old zebrafish. **e)** Trunk cross section at the level indicated by a dashed line in illustration above of 12 months old WT, *desma*$^{-/-}$*;desmb*$^{-/-}$, *desma*$^{-/-}$*;desmb*$^{-/-}$*;fhl2a*$^{-/-}$, *desma*$^{-/-}$*;desmb*$^{-/-}$*;fhl2b*$^{-/-}$ and *desma*$^{-/-}$*;desmb*$^{-/-}$*;fhl2a*$^{-/-}$*;fhl2b*$^{-/-}$ zebrafish immunolabelled with F310 (fast myofibers). **f)** Quantification of F310^{+} myofiber size. **g)** Trunk cross section of 12 months old WT, *desma*$^{-/-}$*;desmb*$^{-/-}$, *desma*$^{-/-}$*;desmb*$^{-/-}$*;fhl2a*$^{-/-}$, *desma*$^{-/-}$*;desmb*$^{-/-}$*;fhl2b*$^{-/-}$ and *desma*$^{-/-}$*;desmb*$^{-/-}$*;fhl2a*$^{-/-}$*;fhl2b*$^{-/-}$ zebrafish immunolabelled with S58 (slow myofibers). **h)** Quantification of S58^{+} myofiber size. Statistical analysis in c, f, h: Two-sided t-tests with Welch correction. Data in violin plots is presented as median (line) and quartiles (dashed line). Abbreviations: A: atrium, BA: bulbus arteriosus, V: ventricle. Scale bar in a 1 cm,b: 500 µm, d: 200 µm, e, g: 100 µm. Schematic images were adapted from https://www.biorender.com.

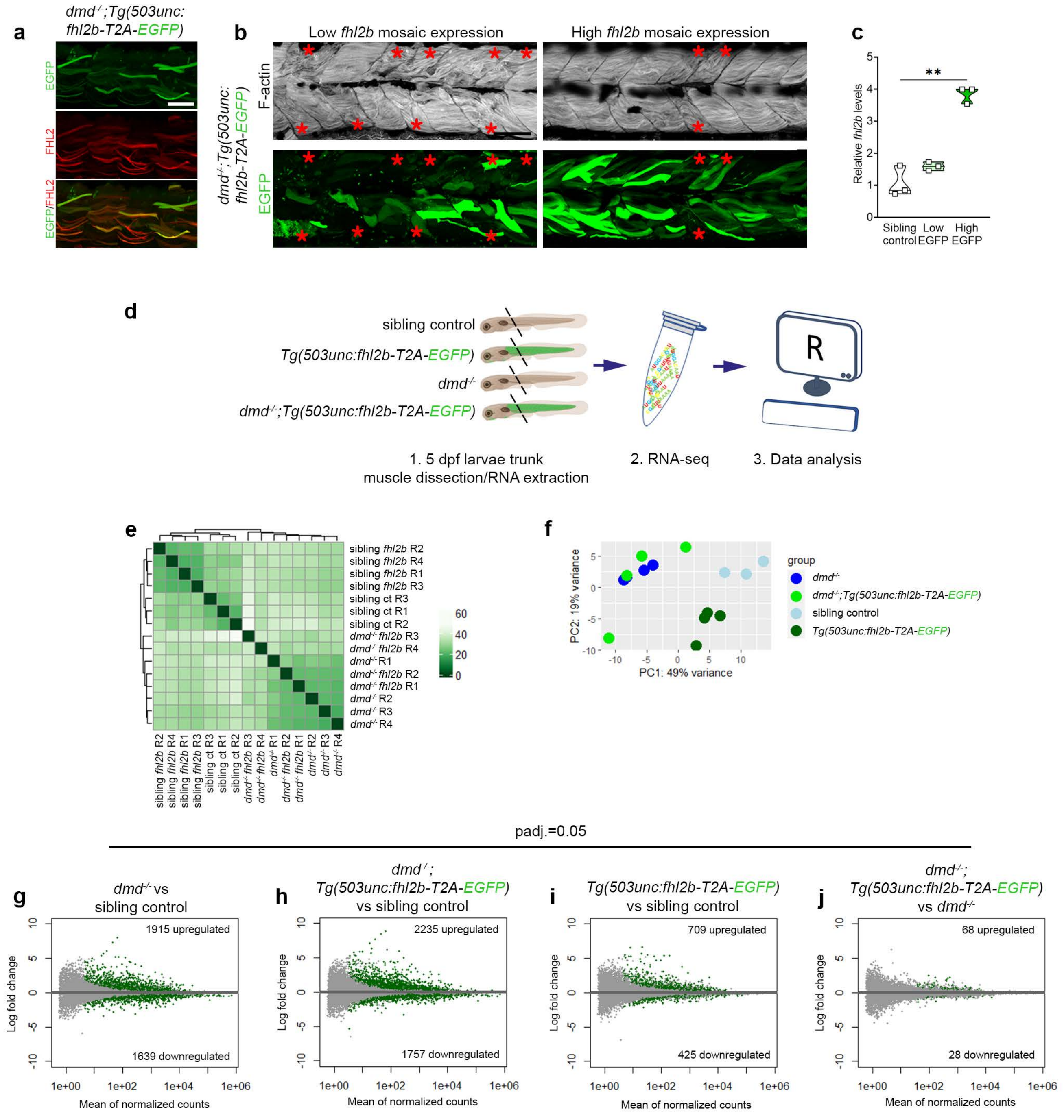
a
dmd-/-;Tg(503unc:
fhl2b-T2A-EGFP)
EGFP
FHL2
EGFP/FHL2
b
Low fhl2b mosaic expression
High fhl2b mosaic expression
dmd-/-;Tg(503unc:
fhl2b-T2A-EGFP)
F-actin
EGFP
c
Relative fhl2b levels
**
Sibling control
Low EGFP
High EGFP
d
sibling control
Tg(503unc:fhl2b-T2A-EGFP)
dmd-/-
dmd-/-;Tg(503unc:fhl2b-T2A-EGFP)
R
1. 5 dpf larvae trunk
muscle dissection/RNA extraction
2. RNA-seq
3. Data analysis
e
sibling fhl2b R2
sibling fhl2b R4
sibling fhl2b R1
sibling fhl2b R3
sibling ct R3
sibling ct R1
sibling ct R2
dmd-/- fhl2b R3
dmd-/- fhl2b R4
dmd-/- R1
dmd-/- fhl2b R2
dmd-/- fhl2b R1
dmd-/- R2
dmd-/- R3
dmd-/- R4
f
PC2: 19% variance
PC1: 49% variance
group
dmd-/-
dmd-/-;Tg(503unc:fhl2b-T2A-EGFP)
sibling control
Tg(503unc:fhl2b-T2A-EGFP)
padj.=0.05
g
dmd-/- vs
sibling control
1915 upregulated
1639 downregulated
h
dmd-/-;
Tg(503unc:fhl2b-T2A-EGFP)
vs sibling control
2235 upregulated
1757 downregulated
i
Tg(503unc:fhl2b-T2A-EGFP)
vs sibling control
709 upregulated
425 downregulated
j
dmd-/-;
Tg(503unc:fhl2b-T2A-EGFP)
vs dmd-/-
68 upregulated
28 downregulated
Log fold change
Mean of normalized counts
1e+00
1e+02
1e+04
1e+06

Supplemental Figure 6. Transcriptomic profiling of *dmd*$^{-/-}$ larval trunk muscle with ectopic *fhl2b* overexpression a) Lateral view of mosaic *Tg(503unc:fhl2b-T2A-EGFP)* zebrafish larvae trunk at 5 dpf immunolabeled with Fhl2 antibodies. **b)** *Tg(503unc:fhl2b-T2A-EGFP)* mosaic zebrafish larvae at 5 dpf with low and high mosaic expression labelled with Phalloidin to visualize F-actin. Asterisk indicates examples of somites with compromised integrity. **c)** Relative level of *fhl2b* in *Tg(503unc:fhl2b-T2A-EGFP)* zebrafish larvae with low and high stable EGFP expression p=0.003. Statistical analysis in: t-tests with Welch correction. Data is presented as median (line) and quartiles (dashed line). **d)** Workflow for RNA-sequencing of 5 dpf sibling control (*dmd*$^{+/-}$, *dmd*$^{+/+}$), *Tg(503unc:fhl2b-T2A-EGFP)*, *dmd*$^{-/-}$ and *dmd*$^{-/-}$*:Tg(503unc:fhl2b-T2A-EGFP)* trunks. Larvae were cut distally to the swim bladder, indicated by the dashed line. **e)** Sample-to-sample distance heatmap and **f)** PCA plot for all four conditions. **g-j)** MA-plots showing differentially expressed genes (DEGs, green dots) for the comparisons **g)** *dmd*$^{-/-}$ *vs* sibling control, **h)** *dmd*$^{-/-}$*:Tg(503unc:fhl2b-T2A-EGFP) vs* sibling control, **i)** *Tg(503unc:fhl2b-T2A-EGFP) vs* sibling control and **j)** *dmd*$^{-/-}$ *vs dmd*$^{-/-}$ *:Tg(503unc:fhl2b-T2A-EGFP)*. Scale bar: 100 µm. Schematic images were adapted from https://www.biorender.com.

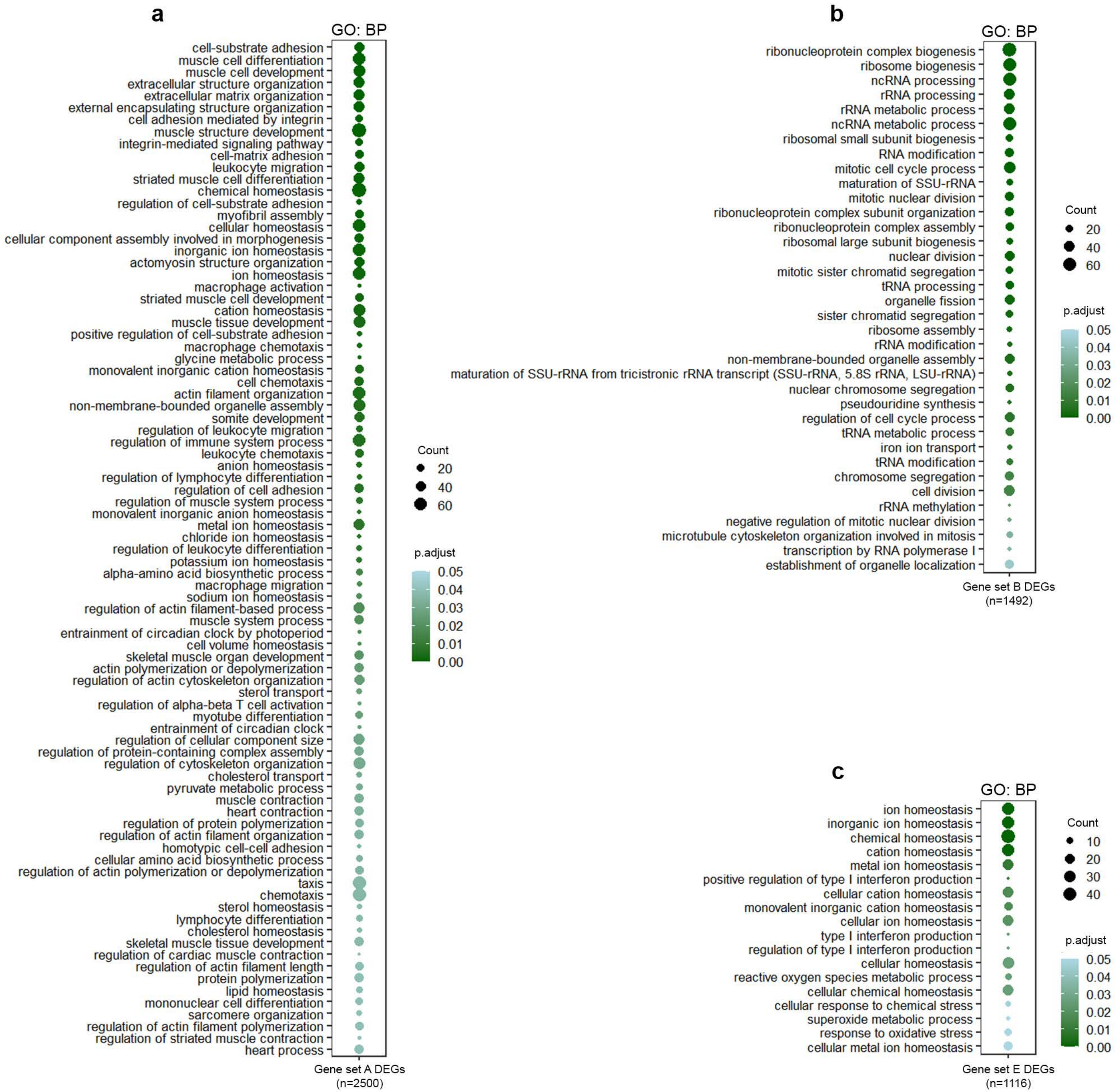
a
GO: BP
cell-substrate adhesion
muscle cell differentiation
muscle cell development
extracellular structure organization
extracellular matrix organization
external encapsulating structure organization
cell adhesion mediated by integrin
muscle structure development
integrin-mediated signaling pathway
cell-matrix adhesion
leukocyte migration
striated muscle cell differentiation
chemical homeostasis
regulation of cell-substrate adhesion
myofibril assembly
cellular homeostasis
cellular component assembly involved in morphogenesis
inorganic ion homeostasis
actomyosin structure organization
ion homeostasis
macrophage activation
striated muscle cell development
cation homeostasis
muscle tissue development
positive regulation of cell-substrate adhesion
macrophage chemotaxis
glycine metabolic process
monovalent inorganic cation homeostasis
cell chemotaxis
actin filament organization
non-membrane-bounded organelle assembly
somite development
regulation of leukocyte migration
regulation of immune system process
leukocyte chemotaxis
anion homeostasis
regulation of lymphocyte differentiation
regulation of cell adhesion
regulation of muscle system process
monovalent inorganic anion homeostasis
metal ion homeostasis
chloride ion homeostasis
regulation of leukocyte differentiation
potassium ion homeostasis
alpha-amino acid biosynthetic process
macrophage migration
sodium ion homeostasis
regulation of actin filament-based process
muscle system process
entrainment of circadian clock by photoperiod
cell volume homeostasis
skeletal muscle organ development
actin polymerization or depolymerization
regulation of actin cytoskeleton organization
sterol transport
regulation of alpha-beta T cell activation
myotube differentiation
entrainment of circadian clock
regulation of cellular component size
regulation of protein-containing complex assembly
regulation of cytoskeleton organization
cholesterol transport
pyruvate metabolic process
muscle contraction
heart contraction
regulation of protein polymerization
regulation of actin filament organization
homotypic cell-cell adhesion
cellular amino acid biosynthetic process
regulation of actin polymerization or depolymerization
taxis
chemotaxis
sterol homeostasis
lymphocyte differentiation
cholesterol homeostasis
skeletal muscle tissue development
regulation of cardiac muscle contraction
regulation of actin filament length
protein polymerization
lipid homeostasis
mononuclear cell differentiation
sarcomere organization
regulation of actin filament polymerization
regulation of striated muscle contraction
heart process
Gene set A DEGs
(n=2500)
Count
20
40
60
p.adjust
0.05
0.04
0.03
0.02
0.01
0.00
b
GO: BP
ribonucleoprotein complex biogenesis
ribosome biogenesis
ncRNA processing
rRNA processing
rRNA metabolic process
ncRNA metabolic process
ribosomal small subunit biogenesis
RNA modification
mitotic cell cycle process
maturation of SSU-rRNA
mitotic nuclear division
ribonucleoprotein complex subunit organization
ribonucleoprotein complex assembly
ribosomal large subunit biogenesis
nuclear division
mitotic sister chromatid segregation
tRNA processing
organelle fission
sister chromatid segregation
ribosome assembly
rRNA modification
non-membrane-bounded organelle assembly
maturation of SSU-rRNA from tricistronic rRNA transcript (SSU-rRNA, 5.8S rRNA, LSU-rRNA)
nuclear chromosome segregation
pseudouridine synthesis
regulation of cell cycle process
tRNA metabolic process
iron ion transport
tRNA modification
chromosome segregation
cell division
rRNA methylation
negative regulation of mitotic nuclear division
microtubule cytoskeleton organization involved in mitosis
transcription by RNA polymerase I
establishment of organelle localization
Gene set B DEGs
(n=1492)
Count
20
40
60
p.adjust
0.05
0.04
0.03
0.02
0.01
0.00
c
GO: BP
ion homeostasis
inorganic ion homeostasis
chemical homeostasis
cation homeostasis
metal ion homeostasis
positive regulation of type I interferon production
cellular cation homeostasis
monovalent inorganic cation homeostasis
cellular ion homeostasis
type I interferon production
regulation of type I interferon production
cellular homeostasis
reactive oxygen species metabolic process
cellular chemical homeostasis
cellular response to chemical stress
superoxide metabolic process
response to oxidative stress
cellular metal ion homeostasis
Gene set E DEGs
(n=1116)
Count
10
20
30
40
p.adjust
0.05
0.04
0.03
0.02
0.01
0.00

Supplemental Figure 7. Gene ontology signatures of *fhl2b* overexpressing larvae. a-c) Gene Ontology terms enriched for the DEGs in the gene sets A, B and E (obtained by the intersection in Fig. 5a, Fig. 6a) are displayed in panels A, B and C, respectively. BP: Biological Process.

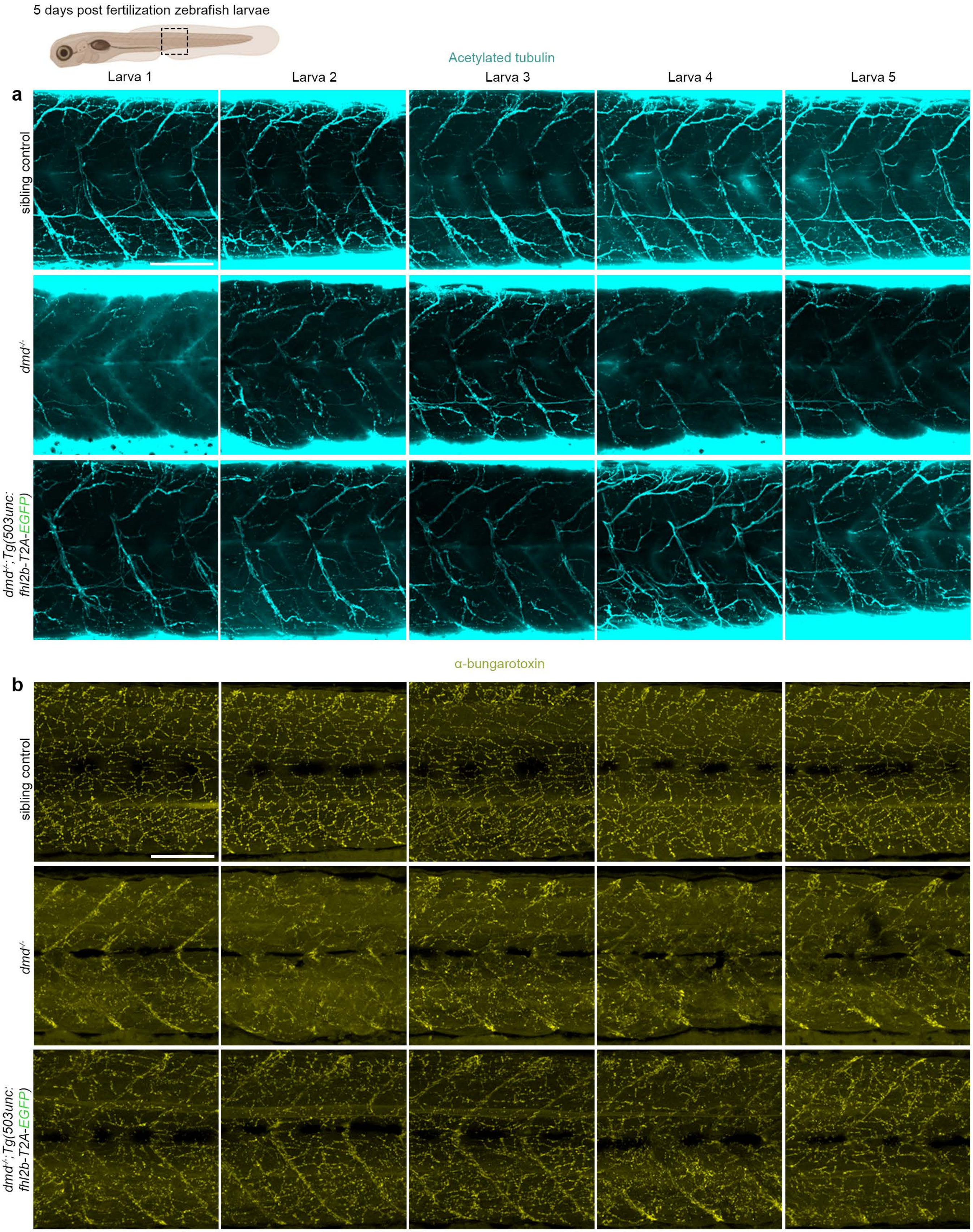

5 days post fertilization zebrafish larvae
Acetylated tubulin
Larva 1
Larva 2
Larva 3
Larva 4
Larva 5
a
sibling control
dmd-/-
dmd-/-;Tg(503unc:
fhl2b-T2A-EGFP)
α-bungarotoxin
b
sibling control
dmd-/-
dmd-/-;Tg(503unc:
fhl2b-T2A-EGFP)

Supplemental Figure 8. *fhl2b* overexpression improves motor neuron axons and NMJ integrity of *dmd*$^{-/-}$ larvae. Examples from 5 sibling control, *dmd*$^{-/-}$ and *dmd*$^{-/-}$*:Tg(503unc:fhl2b-T2A-EGFP)* larvae immunolabeled for **a)** acetylated tubulin and **b)** α-bungarotoxin at 5 dpf, visualizing axons and NMJs, respectively. Area viewed is indicated in illustration above. Scale bar: 100 µm. Schematic images were adapted from https://www.biorender.com.

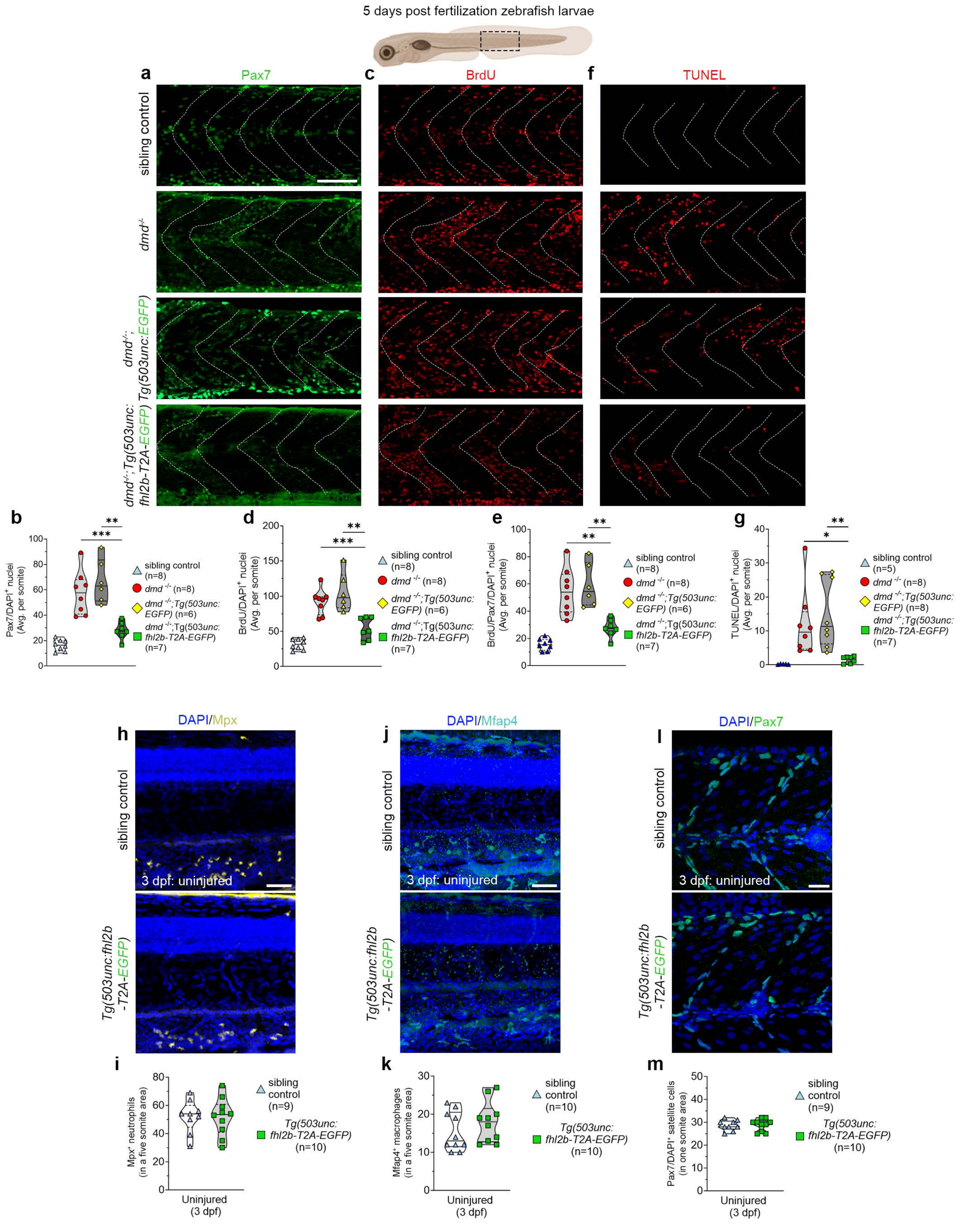
5 days post fertilization zebrafish larvae
a
Pax7
c
BrdU
f
TUNEL
sibling control
dmd-/-
dmd-/-;Tg(503unc:EGFP)
dmd-/-;Tg(503unc:fhl2b-T2A-EGFP)
b
Pax7/DAPI+ nuclei (Avg. per somite)
sibling control (n=8)
dmd -/- (n=8)
dmd -/-;Tg(503unc:EGFP) (n=6)
dmd -/-;Tg(503unc:fhl2b-T2A-EGFP) (n=7)
d
BrdU/DAPI+ nuclei (Avg. per somite)
sibling control (n=8)
dmd -/- (n=8)
dmd -/-;Tg(503unc:EGFP) (n=6)
dmd -/-;Tg(503unc:fhl2b-T2A-EGFP) (n=7)
e
BrdU/Pax7/DAPI+ nuclei (Avg. per somite)
sibling control (n=8)
dmd -/- (n=8)
dmd -/-;Tg(503unc:EGFP) (n=6)
dmd -/-;Tg(503unc:fhl2b-T2A-EGFP) (n=7)
g
TUNEL/DAPI+ nuclei (Avg. per somite)
sibling control (n=5)
dmd -/- (n=8)
dmd -/-;Tg(503unc:EGFP) (n=8)
dmd -/-;Tg(503unc:fhl2b-T2A-EGFP) (n=7)
h
DAPI/Mpx
j
DAPI/Mfap4
l
DAPI/Pax7
sibling control
Tg(503unc:fhl2b-T2A-EGFP)
3 dpf: uninjured
i
Mpx+ neutrophils (in a five somite area)
sibling control (n=9)
Tg(503unc:fhl2b-T2A-EGFP) (n=10)
Uninjured (3 dpf)
k
Mfap4+ macrophages (in a five somite area)
sibling control (n=10)
Tg(503unc:fhl2b-T2A-EGFP) (n=10)
Uninjured (3 dpf)
m
Pax7/DAPI+ satellite cells (in one somite area)
sibling control (n=9)
Tg(503unc:fhl2b-T2A-EGFP) (n=10)
Uninjured (3 dpf)

Supplemental Figure 9. *Fhl2b* overexpression improves muscle integrity in *dmd*$^{-/-}$ larvae. Lateral view of sibling control (*dmd*$^{+/+}$, *dmd*$^{+/-}$), *dmd*$^{-/-}$, *dmd*$^{-/-}$*:Tg(503unc:EGFP)* and *dmd*$^{-/-}$*:Tg(503unc:fhl2b-T2A-EGFP)* 5 dpf larvae immunolabeled for **a)** Pax7 showed **b)** a significantly decreased numbers of Pax7^{+} cells in *dmd*$^{-/-}$*:Tg(503unc:fhl2b-T2A-EGFP)* as compared to *dmd*$^{-/-}$ (p=0.0009) and *dmd*$^{-/-}$*:Tg(503unc:EGFP)* (p=0.0018). **c)** Incorporation of BrdU (24 h pulse) showed **d)** a significant decrease in proliferating cell numbers in *dmd*$^{-/-}$*:Tg(503unc:fhl2b-T2A-EGFP)* as compared to *dmd*$^{-/-}$ (p=0.0009) and *dmd*$^{-/-}$*:Tg(503unc:EGFP)* (p=0.0039) as well as **e)** decreased levels of proliferating Pax7^{+} cells in *dmd*$^{-/-}$*:Tg(503unc:fhl2b-T2A-EGFP)* as compared to *dmd*$^{-/-}$ (p=0.0017) and *dmd*$^{-/-}$*:Tg(503unc:EGFP)* (p=0.0035). **f)** TUNEL labeling showed **g)** a significant decrease in cell death in *dmd*$^{-/-}$*:Tg(503unc:fhl2b-T2A-EGFP)* as compared to *dmd*$^{-/-}$ (p=0.0211) and *dmd*$^{-/-}$*:Tg(503unc:EGFP)* (p=0.0065). **h)** Immunolabeling of neutrophils using Mpx antibodies in uninjured 3 dpf sibling controls and *Tg(503unc:fhl2b-T2A-EGFP)*. **i)** Quantifications of DAPI/Mpx positive neutrophils in five somites in 3 dpf sibling controls and *Tg(503unc:fhl2b-T2A-EGFP)*. **j)** Immunolabeling of macrophages using Mfap4 antibodies in uninjured 3 dpf sibling controls and *Tg(503unc:fhl2b-T2A-EGFP)*. **k)** Quantifications of DAPI/Mfap4 positive macrophages in five somites in 3 dpf sibling controls and *Tg(503unc:fhl2b-T2A-EGFP)*. **l)** Immunolabeling of satellite cells using Pax7 antibodies in uninjured 3 dpf sibling controls and *Tg(503unc:fhl2b-T2A-EGFP)*. **m)** Quantifications of DAPI/Pax7 positive satellite cells in one somite in 3 dpf sibling controls and *Tg(503unc:fhl2b-T2A-EGFP)*. Statistical analysis in b, d, e, g, i, k, m: Two-sided t-tests with Welch correction. Data in violin plots (b, d, e, g, i, k, m) is presented as median (line) and quartiles (dashed line). Schematic images were adapted from https://www.biorender.com.
