# Supplementary material for: fhl2b mediates extraocular muscle protection in zebrafish models of muscular dystrophies and its ectopic expression ameliorates affected body muscles
Source: Nat Commun. 2024 Mar 2;15:1950. doi: 10.1038/s41467-024-46187-x (PMC10908798; doi:10.1038/s41467-024-46187-x)
Supplement: Supplementary file 3 — Description of Additional Supplementary Files [file 41467_2024_46187_MOESM3_ESM.pdf]

Description of additional Supplementary files.

File name: Supplementary Data 1

Description: Sample and mapping information regarding EOM and trunk muscle RNAsequencing experiments in WT and desma-/-; desmb-/- adult zebrafish at five and 20 months of age. Furthermore, this table includes full DEG lists of the eight different comparisons used in the study.

File name: Supplementary Data 2

Description: This table includes filtered DEG lists of the eight different comparisons used in

the study including only the muscle related DEGs for each comparison.

File name: Supplementary Data 3

Description: Sample and mapping information regarding trunk muscle RNA-sequencing experiments in sibling controls (dmd+/+ , dmd+/-), sibling controls with fhl2b overexpression (Tg(503unc:fhl2b-T2A-EGFP)), dmd-/- and dmd-/- ;Tg(503unc:fhl2b-T2A-EGFP) larvae. Furthermore, this table includes full DEG lists of the four comparisons used in this study.

File name: Supplementary Data 4

Description: Full list of the antibodies used in this study.

File name: Supplementary Data 5

Description: Full list of the qPCR probe sequences used in this study
