# Supplementary material for: fhl2b mediates extraocular muscle protection in zebrafish models of muscular dystrophies and its ectopic expression ameliorates affected body muscles
Source: Nat Commun. 2024 Mar 2;15:1950. doi: 10.1038/s41467-024-46187-x (PMC10908798; doi:10.1038/s41467-024-46187-x)
Supplement: Supplementary file 9 — Reporting Summary [file 41467_2024_46187_MOESM9_ESM.pdf]

nature portfolio

Corresponding author(s): Fatima Pedrosa Domellöf & Jonas von Hofsten

Last updated by author(s): Feb 12, 2024

Reporting Summary

Nature Portfolio wishes to improve the reproducibility of the work that we publish. This form provides structure for consistency and transparency in reporting. For further information on Nature Portfolio policies, see our Editorial Policies and the Editorial Policy Checklist.

Statistics

For all statistical analyses, confirm that the following items are present in the figure legend, table legend, main text, or Methods section.

n/a	Confirmed	
☐	☒	The exact sample size (*n*) for each experimental group/condition, given as a discrete number and unit of measurement
☐	☒	A statement on whether measurements were taken from distinct samples or whether the same sample was measured repeatedly
☐	☒	The statistical test(s) used AND whether they are one- or two-sided<br>*Only common tests should be described solely by name; describe more complex techniques in the Methods section.*
☒	☐	A description of all covariates tested
☐	☒	A description of any assumptions or corrections, such as tests of normality and adjustment for multiple comparisons
☐	☒	A full description of the statistical parameters including central tendency (e.g. means) or other basic estimates (e.g. regression coefficient) AND variation (e.g. standard deviation) or associated estimates of uncertainty (e.g. confidence intervals)
☐	☒	For null hypothesis testing, the test statistic (e.g. F, t, r) with confidence intervals, effect sizes, degrees of freedom and P value noted<br>*Give P values as exact values whenever suitable.*
☒	☐	For Bayesian analysis, information on the choice of priors and Markov chain Monte Carlo settings
☒	☐	For hierarchical and complex designs, identification of the appropriate level for tests and full reporting of outcomes
☒	☐	Estimates of effect sizes (e.g. Cohen's *d*, Pearson's *r*), indicating how they were calculated

Our web collection on statistics for biologists contains articles on many of the points above.

Software and code

Policy information about availability of computer code

Data collection	-Next Generation Sequencing: NovaSeq 6000 System, Illumina.<br>-Image Collection: Nikon A1 confocal microscope and a Nikon SMZ18 microscope (birefringence) utilizing the NIS-Elements software (v5.02.03).<br>-Zebrafish larvae swimming: Viewpoint Zebrabox system, utilizing the Zebralab software (v5.15.0.40).<br>-qPCR: VIIA-7 Real Time PCR system using the ViiA-7 Software (v1.1).<br>-Western blot: Odyssey Fc Dual-Mode Imaging System using the Image Studio (v5.2) software.
Data analysis	-Publicly avaliable tools used in this study:<br>STAR (2.7.6a)<br>-Open source tools available via Bioconductor (3.11-3.16), R environment (v.4.0.0-v.4.2.3)<br>DESeq2 (1.30.1)<br>ComplexHeatmap (2.6.2)<br>clusterProfiler (4.0.5)<br>enrichplot (1.18)<br>ggplot2 (3.4.3)<br>org.Dr.eg.db (3.16)<br><br>Image management and quantification:<br>Microsoft Excel (Office 365)<br>Cellprofiler (4.2.5)<br>ImageJ (1.54, Fiji, Labkit)

Imaris (10.1, FilamentTracer, Surface)
GraphPad Prism 10.0
Images were prepared in Adobe Photoshop CS6.

For manuscripts utilizing custom algorithms or software that are central to the research but not yet described in published literature, software must be made available to editors and reviewers. We strongly encourage code deposition in a community repository (e.g. GitHub). See the Nature Portfolio guidelines for submitting code & software for further information.

Data

Policy information about availability of data

All manuscripts must include a data availability statement. This statement should provide the following information, where applicable:

- Accession codes, unique identifiers, or web links for publicly available datasets
- A description of any restrictions on data availability
- For clinical datasets or third party data, please ensure that the statement adheres to our policy

The RNA-sequencing data sets utilized in this study have been deposited in GEO (Gene Expression Omnibus) under the accession number GSE242137, including the subseries:
- GSE242134 [20 months EOM and trunk]
- GSE242135 [5dpf trunk]
- GSE242136 [5 months EOM and trunk]

and were released to the public on 2024-02-08: https://www.ncbi.nlm.nih.gov/geo/query/acc.cgi?acc=GSE242137

All RNA-sequencing data generated in this study has been mapped to the zebrafish genome (GRCz11).

Research involving human participants, their data, or biological material

Policy information about studies with human participants or human data. See also policy information about sex, gender (identity/presentation), and sexual orientation and race, ethnicity and racism.

Reporting on sex and gender	Four men and one woman ages 47-80. Sex was not considered in this study and all samples showed similar results in our experiments.
Reporting on race, ethnicity, or other socially relevant groupings	N/A
Population characteristics	A total of ten EOM muscle samples were obtained at autopsy from five donors four men and one woman ages 47-80 with no previous known neuromuscular disease.
Recruitment	Human material was collected soley from diseased individuals who, before the time of death, had agreed to donate their tissue and organs to medical research.
Ethics oversight	The study was conducted with the approval of the Regional Ethical Review Board in Umeå (Dnr: 2010-373-31M) and was completed in accordance with the principles of the Declaration of Helsinki.

Note that full information on the approval of the study protocol must also be provided in the manuscript.

Field-specific reporting

Please select the one below that is the best fit for your research. If you are not sure, read the appropriate sections before making your selection.

☒ Life sciences ☐ Behavioural & social sciences ☐ Ecological, evolutionary & environmental sciences

For a reference copy of the document with all sections, see nature.com/documents/nr-reporting-summary-flat.pdf

Life sciences study design

All studies must disclose on these points even when the disclosure is negative.

Sample size	No statistical methods were used to predetermine sample size. For RNA-sequencing experiments (5 months old zebrafish), each analyzed sample contained a pool of tissue, either EOMs or trunk muscle of 30 fish per biological replica. The number of zebrafish used in each group was that necessary to achieve the required amount of RNA needed to perform RNA sequencing. Three biological replicates per group of each genotype and tissue, WT/desma; desmb KO EOMs and trunk muscles were used. At 20 months old zebrafish 12 fish per biological replica was used. The number of zebrafish used in each group was that necessary to achieve the required amount of RNA needed to perform RNA-sequencing. The difference in the number of fish used at 20 months compared to at five months depends on differences in fish size. Three biological replicates per group of WT/desma; desmb KO EOMs and trunk muscles were used. For RNA-sequencing of five dpf zebrafish, 12 larval trunk muscle samples per individual group were used, four biological replicates per group of sibling control/dmd KO/ sibling; Tg(503unc:fhl2b-T2A-EGFP)/dmd KO;Tg(503unc:fhl2b-T2A-EGFP). For details please refer to the methods section under the subtitle of RNA

sequencing and analysis. For all other experiments, the number of biological replicates used in each experiment is indicated in the figure legends. The number of groups used in each experiment were the standard amount required to perform relevant statistical analysis.

Data exclusions

For the 5 dpf RNA-seq analysis, sibling control sample 3 was excluded from the analysis as it showed ~50% variance from other sibling control samples on the PC1 axis and did not cluster to any samples in sample to sample distance plots in the multiQC report.

Replication

RNA seq libraries were sequenced on a Novaseq 6000 Sequencing system (Illumina, Inc.) and an average of ~78.5 million 150 paired end reads per library was obtained. RNA seq was performed in triplicates or quadruplicates. For details refer to Supplement Tables 1, 2 and 3. All major conclusions drawn from RNA-sequencing experiments were confirmed by immunofluorescence and tested in vivo. At least 2-3 independent experiments were carried out and representative examples were shown. All attempts at data replication were successful. Please refer to figure legends for the exact number of zebrafish used in each experiment.

Randomization

Randomization was applied to our study. However, all biological samples were treated or analyzed in the same manner.

Blinding

All experiments were performed blind, with the exception of experiments where genotype was evident based on phenotype.
qPCR of fhl2 mutants were not blinded as this required genotyping and pooling of embryos with the same genotype and the same investigator performed the experiments.
Studies of dmd-/- larvae with or without fhl2b overexpression was not blinded as these embryos had obvious differences based on genotype.

Reporting for specific materials, systems and methods

We require information from authors about some types of materials, experimental systems and methods used in many studies. Here, indicate whether each material, system or method listed is relevant to your study. If you are not sure if a list item applies to your research, read the appropriate section before selecting a response.

Materials & experimental systems

n/a	Involved in the study
☐	☒ Antibodies
☒	☐ Eukaryotic cell lines
☒	☐ Palaeontology and archaeology
☐	☒ Animals and other organisms
☒	☐ Clinical data
☒	☐ Dual use research of concern
☒	☐ Plants

Methods

n/a	Involved in the study
☒	☐ ChIP-seq
☒	☐ Flow cytometry
☒	☐ MRI-based neuroimaging

Antibodies

Antibodies used

Antibody Application Clonality Dilution Source Productnr/clone lot#
F310 IHC Mouse 1:10 DSHB AB_531863 lot: 12-27-18
S58 IHC Mouse 1:10 DSHB AB_528377 lot: 8-12-21
Pax7 IHC Mouse 1:10 DSHB AB_528428 lot: 7-19-20
SV2 IHC mouse 1:10 DSHB AB_2315387 lot: 5-23-19
Desmin IHC Rabbit 1:100 Abcam ab15200 lot: GR3323784-2
Laminin IHC Rabbit 1:100 Sigma-Aldrich L9393 lot: 0000141521
Laminin-2 IHC Rat 1:100 Enzo Life Sciences ALX-804-190 lot:L26855
PCNA IHC Rabbit 1:100 Sigma-Aldrich SAB2701819 lot: GT40541
Acetylated Tubulin IHC Rabbit 1:100 Sigma-Aldrich T7451 lot: 109M4831V
FHL2 IHC/WB Rabbit 1:100/1:1000 Sigma-Aldrich HPA005922 lot:A60689
FHL2 IHC WB mouse 1:1000 Medical and Biological laboratory K0055-3 lot:014
MPX IHC Rabbit 1:100 GeneTex GTX128379 lot: 44434
MFAP4 IHC Rabbit 1:100 GeneTex GTX132692 lot: 1014275-1
BrdU-555 IHC Directly conjugated 1:500 BD Biosciences 560210/clone 3D4 lot: 8241971
GAPDH WB Mouse 1:2000 Abcam AB8245 lot:GR3438148-1
Flourescin AffiniPure™ donkey anti-rat 1:100 Jackson ImmunoResearch 715-095-150 lot: 28196
Alexa Fluor 488 donkey anti-rabbit IgG 1:300 Invitrogen, Molecular probes A-21206 lot: 157934
Alexa Fluor 488 donkey anti-mouse IgG 1:300 Invitrogen, Molecular probes A-21202 lot: 138499
Rhodamine Red™-X donkey anti-rabbit 1:500 Jackson ImmunoResearch AB_2340613 lot: 145693
Rhodamine Red™-X donkey anti-mouse 1:500 Jackson ImmunoResearch AB_2340832 lot: 162591
Alexa Fluor 647 goat anti-mouse IgG 1:300 Invitrogen, Molecular probes A32795 lot: 2379464
Alexa Fluor 647 goat anti-rabbit IgG 1:300 Invitrogen, Molecular probes A32787 lot: 2179230
Alexa Fluor 488 Phalloidin Directly conjugated 1:100 Invitrogen, Molecular probes A12379 lot: 41D1-2
Phalloidin rhodamine Directly conjugated 1:100 Invitrogen, Molecular probes R415 lot: 899165
Alexa Fluor 647 Phalloidin Directly conjugated 1:100 Invitrogen, Molecular probes A22287 lot: 1750839
DAPI IHC Directly conjugated 1:500 Sigma-Aldrich D9542
Anti-rabbit IgG, HRP-linked secondary antibody 1:2000 Cell Signaling 7074
Anti-mouse IgG HRP-linked secondary antibody 1:2000 Cell Signaling 7076

Validation

F310 - https://dshb.biology.uiowa.edu/F310

Validation	S58 - https://dshb.biology.uiowa.edu/S58<br>Pax7 - https://dshb.biology.uiowa.edu/PAX7<br>SV2 - https://dshb.biology.uiowa.edu/SV2<br>Desmin - https://www.abcam.com/en-se/products/primary-antibodies/desmin-antibody-cytoskeleton-marker-ab15200<br>Laminin - https://www.sigmaaldrich.com/SE/en/product/sigma/l9393<br>Laminin-2 - https://www.enzolifesciences.com/ALX-804-190/laminin-2-alpha-2-chain-monoclonal-antibody-4h8-2/<br>PCNA - https://www.sigmaaldrich.com/SE/en/product/sigma/sab2701819<br>Acetylated Tubulin - https://www.sigmaaldrich.com/SE/en/product/sigma/t7451<br>FHL2 - https://www.sigmaaldrich.com/SE/en/product/sigma/hpa005922<br>FHL2 - https://www.mblintl.com/products/k0055-3/<br>MPX - https://www.genetex.com/Product/Detail/Mpx-antibody/GTX128379<br>MFAP4 - https://www.genetex.com/Product/Detail/Mfap4-antibody/GTX132692<br>GAPDH - https://www.abcam.com/en-se/products/primary-antibodies/gapdh-antibody-6c5-loading-control-ab8245<br>BrdU - https://www.bdbiosciences.com/en-us/products/reagents/fluorchrome-conjugated-antibodies/bd-pharmingen-alexa-fluor-555-ex-max-555-nm-em-max-580-nm/alexa-fluor-555-mouse-anti-brdu.560210

Animals and other research organisms

Policy information about studies involving animals; ARRIVE guidelines recommended for reporting animal research, and Sex and Gender in Research

Laboratory animals	Zebrafish larvae (Danio rerio) and adult fish were maintained from AB WT. Mutant lines used were desma:umu10, desmb:umu11, obscnb:umu16, plecb:umu25, fhl2a:umu32, fhl2b:umu33 and sapje:t222a.Transgenic lines used were Tg(mylz2:EGFP):i135, Tg(smyhc1:tdTomato):i261, Tg(503unc:EGFP):umu37 and Tg(503unc:fhl2b-T2A-EGFP):umu34. Ages of zebrafish were predominantly 3-5 dpf, 5 months, 12 months or 20-24 months of age.<br><br>Mixed WT mouse lines were used for western blot, eight weeks old: 129/Sv:CBA/J:C57BL/6J:DBA2/J and for immunohistochemistry four weeks old: 129/Sv:CBA/J:C57BL/6J:DBA2/J.
Wild animals	The study did not include any wild animals.
Reporting on sex	Zebrafish and mice tissue from both sexes were used in this study. A large portion of the study was performed on zebrafish larvae at time points in which sex is not yet genetically determined (<30 dpf).
Field-collected samples	The study did not involve field-collected samples.
Ethics oversight	All zebrafish animal experiments were ethically approved by the Regional Ethics Committee at the court of Appeal of Northern Norrlands Umeå djurförsöksetiska nämnd, Dnr: A6 2020.<br><br>Leftover mouse tissue from mice that were used in terminal experiments performed by other researchers was kindly donated by Leif Carlsson, Umeå University, approved by the Animal Review Board at the Court of Appeal of Northern Norrland in Umeå Dnr: A22-2023.<br><br>A total of ten EOM muscle samples were obtained at autopsy from five human donors (four men and one woman, ages 47-80) who, when alive, had consented to donate their eyes and other tissues post-mortem for transplantation and research purposes, according to Swedish law, with no previous known neuromuscular disease with the approval of the Regional Ethical Review Board in Umeå (Dnr: 2010-373-31M), in accordance with the principles of the Declaration of Helsinki.

Note that full information on the approval of the study protocol must also be provided in the manuscript.

Plants

Seed stocks	N/A
Novel plant genotypes	N/A
Authentication	N/A
